# Association between Family History Risk Categories and Prevalence of Diabetes in Chinese Population

**DOI:** 10.1371/journal.pone.0117044

**Published:** 2015-02-09

**Authors:** Jinping Zhang, Zhaojun Yang, Jianzhong Xiao, Xiaoyan Xing, Juming Lu, Jianping Weng, Weiping Jia, Linong Ji, Zhongyan Shan, Jie Liu, Haoming Tian, Qiuhe Ji, Dalong Zhu, Jiapu Ge, Li Chen, Xiaohui Guo, Zhigang Zhao, Qiang Li, Zhiguang Zhou, Lixiang Lin, Na Wang, Wenying Yang, for the China National Diabetes and Metabolic Disorders Study Group

**Affiliations:** 1 Department of Endocrinology, China–Japan Friendship Hospital, Beijing, China; 2 Department of Endocrinology, Chinese People's Liberation Army General Hospital, Beijing, China; 3 Department of Endocrinology, Sun Yat-sen University Third Hospital, Guangzhou, China; 4 Department of Endocrinology, Shanghai Jiaotong University Affiliated Sixth People's Hospital, Shanghai, China; 5 Department of Endocrinology, Peking University People's Hospital, Beijing, China; 6 Department of Endocrinology, First Affiliated Hospital, Chinese Medical University, Liaoling, China; 7 Department of Endocrinology, Shanxi Province People's Hospital, Taiyuan, Shanxi, China; 8 Department of Endocrinology, West China Hospital, Sichuan University, Chengdu, Sichuan, China; 9 Department of Endocrinology, Xijing Hospital, Fourth Military Medical University, Xi'an, Shanxi, China; 10 Department of Endocrinology, Affiliated Drum Tower Hospital of Nanjing University Medical School, Nanjing, Jiangsu, China; 11 Department of Endocrinology, Xinjiang Uygur Autonomous Region's Hospital, Urmqi, Xinjiang, China; 12 Department of Endocrinology, Qilu Hospital, Qilu Hospital of Shandong University, Jinan, Shandong, China; 13 Department of Endocrinology, Peking University First Hospital, Beijing, China; 14 Department of Endocrinology, Henan Province People's Hospital, Zhengzhou, Henan, China; 15 Department of Endocrinology, Second Affiliated Hospital of Harbin Medical University, Harbin, Heilongjiang, China; 16 Department of Endocrinology, Xiangya Second Hospital, Changsha, Hunan, China; 17 Department of Endocrinology, Fujian Provincial Hospital, Fuzhou, Fujiang, China; Old Dominion University, UNITED STATES

## Abstract

**Aim:**

To investigate the association between different family history risk categories and prevalence of diabetes in the Chinese population.

**Methods:**

The family history of diabetes was obtained from each subject, and an oral glucose tolerance test was performed for measuring the fasting and postload glucose and insulin levels based on a national representative cross-sectional survey of 46,239 individuals (age ≥ 20 years) in the 2007–2008 China National Diabetes and Metabolism Disorders Study. The family history risk categories of diabetes were high, moderate, and average (FH2 and FH1: at least two generations and one generation of first-degree relatives with diabetes, respectively; FH0: no first-degree relatives with diabetes).

**Results:**

The age- and gender-adjusted prevalence rates of diabetes were 32.7% (95% confidence interval (CI): 26.4–39.7%) in FH2, 20.1% (95% CI: 18.2–22.1%) in FH1, and 8.4% (95% CI: 7.9–8.9%) in FH0 (P < 0.0001). The calculated homeostatic model assessment-estimated insulin resistance (HOMA-IR), Matsuda insulin sensitivity index (ISI), and insulinogenic index (ΔI30/ΔG30) values showed significant trending changes among the three risk categories, with the most negative effects in FH2. Multivariate logistic regression analysis showed that the odds ratios of having diabetes were 6.16 (95% CI: 4.46–8.50) and 2.86 (95% CI: 2.41–3.39) times higher in FH2 and FH1, respectively, than in FH0 after adjustment for classical risk factors for diabetes.

**Conclusions:**

Family history risk categories of diabetes have a significant, independent, and graded association with the prevalence of this disease in the Chinese population.

## Introduction

Diabetes mellitus is a major noncontagious disease that has become a serious threat to human health. According to the latest estimates of the International Diabetes Federation, the prevalence of diabetes worldwide has reached 8.3%, and approximately 382 million adults have had diabetes, and 80% of these adults are present in medium and low income countries with scarce health resources [1].

In China, the latest data have shown that prevalence of diabetes in adults aged 20 years or older has increased to an alarming 9.7% [2]. If the American Diabetes Association standard (glycated hemoglobin ≥ 6.5%) is included in the diagnostic criteria for diabetes, then the prevalence of diabetes in the Chinese population reaches 11.6% [3]. The population size of Chinese patients with diabetes is estimated to be 92.4–98.4 million [1,2]; of which, approximately 60% patients have not yet been diagnosed [2]. Due to the scarcity and uneven distribution of health resources in China, effective and targeted screening of high-risk groups and early treatment of diabetes are significant factors for preventing the late complications of this disease.

The etiology of diabetes involves complex genetic and environmental factors[4,5]. A large number of epidemiological studies have indicated that the family history of diabetes, advanced age, obesity (especially abdominal obesity), physical inactivity, and abnormal lipid metabolism are independent risk factors for diabetes. Patients with a positive family history of diabetes experience 3- to 4-fold higher risk of developing this disease than those with a negative family history of diabetes[2,3,6–8], and the risk of diabetes increases with the number of affected relatives [6–9]. Other studies have shown that the level of risk of diabetes is related to the nature of relationship with the affected relatives. For instance, mothers with diabetes contribute more than fathers with diabetes to the risk of type 2 diabetes in the offspring [8], and the first-degree relatives with diabetes are associated with a higher family history risk category than that of second-degree relatives with diabetes [9].

The above findings on the association between the family history risk categories and the prevalence of diabetes were mainly obtained from the European and American populations[6–9]. However, a study on the South Korean population shows no evidence that the offspring of mothers with diabetes have a higher risk of diabetes than other groups [10]. The familial aggregation of diabetes is believed to be the result of combined action by genetic factors and similar environmental factors shared among family members (e.g., diet habits and sedentary lifestyle) [11]. Therefore, it is necessary to explore the association between the family history risk categories and the prevalence of diabetes among different types of ethnic groups to provide a reference for the highly effective identification of high-risk groups of this disease.

In China, two epidemiological studies on diabetes nationwide have been conducted, which show that a positive family history of diabetes is an independent risk factor for this disease in the Chinese population [2,12]. In the 2007–2008 China National Diabetes and Metabolic Disorders Study (DMS), all subjects underwent a standard 75-g oral glucose tolerance test (OGTT) with blood glucose and insulin measurements. Additionally, the prevalence of diabetes in the first-degree relatives of the subjects was surveyed. In the present study, an in-depth analysis on the association among the family history risk categories of diabetes and insulin secretion, insulin resistance, and prevalence of diabetes was carried out using data from the 2007–2008 DMS survey of Chinese adults aged 20 years or older.

## Materials and Methods

### Study subjects

From June 2007 to May 2008, the nationwide DMS was carried out using a complex multistage stratified sampling method. Representative regions (including 152 urban districts and 112 rural villages) were selected across the country by considering the geographical distribution, economic development, and level of urbanization. Residents aged 20 years and older and living locally for 5 years or more were randomly selected from each region. A total of 46,239 subjects (18,419 men and 27,820 women) with mean age of 44.9 years completed the study. Detailed sampling methods can be found in a previous description in the literature [2].

### Ethics Statement

The study program was reviewed and approved by the Clinical Research Ethics Committee of China-Japan Friendship Hospital (No. 2007–026). All the subjects had signed informed consent forms prior to the collection of data and other tests.

### Physical examination and questionnaire survey

Trained and qualified physicians conducted the physical examination of all the subjects according to standard methods of the study program, including the measurement of body height, weight, waist and hip circumferences, blood pressure, and heart rate [2]. The body height and weight of the patients were measured with the patients wearing only underwear and shoes, with an accuracy of 0.1 cm and 0.1 kg, respectively. The body mass index (BMI) was calculated by dividing the body weight (kg) by the square of body height (m). The waist circumference was defined as the minimal horizontal circumference along the connection between the subcostal margin and the iliac crest, and the hip circumference was defined as the maximal horizontal circumference between the waist and the hips. Central obesity was defined as a waist circumference of ≥ 90 cm in males and ≥ 85 cm in females [13,14]. After 10–15 min of rest at sitting position, the blood pressure was measured twice at a 30-s interval in the right upper arm using a mercury sphygmomanometer; the arithmetic mean of the two measurements was taken as the blood pressure value.

Data were collected in health stations or community clinics near the residence of the subjects. During an on-site survey, a standard questionnaire was completed by trained, qualified physicians and nurses by face-to-face method. Each subject was queried about his/her lifestyle, previous maximal weight, history of diabetes, time of diagnosis of diabetes, and history of medication, as well as family histories of coronary heart disease, stroke, and diabetes.

### Family history of diabetes

The DMS questionnaire survey collected information about the presence or absence of diabetes in the first-degree relatives of the subjects (including father, mother, siblings, and children). If the answer was “yes,” then the subject was considered to have a positive family history of diabetes and further had to specify the nature of relationship with those who have diabetes. If the answer was “no,” then the subject was considered to have a negative family history of diabetes. In the case of “unknown,” “refuse to answer,” or “forget to ask,” the subject was excluded from the following analysis because of the uncertainty regarding the family history of diabetes. In accordance with the family history of diabetes, the risk categories of disease were defined as follows: (1) Average: no first-degree relatives with diabetes (FH0); (2) moderate: only one generation of first-degree relatives with diabetes (FH1); and (3) high: at least two generations of first-degree relatives with diabetes (FH2).

### OGTT or standard meal test

The subjects were requested to avoid excessive exercise or diet control for at least 3 days prior to the test. After fasting overnight for 10–12 h, blood specimens were collected using a vacuum tube with sodium fluoride for determining fasting plasma glucose, lipid profile, and insulin. Then, the subjects with no history of diabetes received an oral dose (75 g) of glucose solution (25%), and those previously diagnosed with diabetes had a standard meal containing 80 g of carbohydrate. Blood specimens were collected at 30 and 120 min after the loading using a vacuum tube with sodium fluoride for determining postload glucose and insulin.

Level of blood glucose was measured using the hexokinase enzymatic method, and lipid profile was measured enzymatically in local laboratories that are recognized by a national or provincial quality control system. For analyzing the levels of insulin, blood specimens were immediately centrifuged at 4°C, and the serum specimens obtained were transported to the Central Laboratory of Endocrinology at the China-Japan Friendship Hospital via the cold chain transportation system, and then stored at -80°C until used. Level of serum insulin was measured using radioactive immunoassay (Beckman insulin kit, Prague, Czech Republic) in an authorized laboratory by certified technicians from China National Nuclear Corporation (Beijing, China). The diagnostic criteria for diabetes, impaired fasting glucose (IFG), and impaired glucose tolerance (IGT) were based on the 1999 World Health Organization Criteria [15]. Prediabetes refers to IFG or IGT, and diabetes and prediabetes are collectively referred to as dysglycemia.

### Calculation of β-cell function and insulin resistance indices

The β-cell function is indicated by two indices: (1) The insulinogenic index (ΔI30/ΔG30) refers to the insulin increment at 30 min after glucose loading relative to fasting serum insulin divided by the plasma glucose increment at 30 min, i.e., ΔI30/ΔG30 = (INS30- FINS)/(PG30 - FPG), where INS30 and PG30 are serum insulin and plasma glucose levels at 30 min after OGTT, respectively; and FINS and FPG are the fasting serum insulin and fasting plasma glucose levels, respectively [16] and (2) corrected insulin response (CIR), i.e., (100 × insulin [mU/L] at 30 min)/(glucose [mmol/L] at 30 min) × (glucose [mmol/L] at 30 min—3.89 mmol/L) [17].

The insulin resistance was indicated by homeostatic model assessment-estimated insulin resistance (HOMA-IR, FINS × FPG/22.5) [18] and the Matsuda insulin sensitivity index, ISI = 10,000/√(fasting plasma glucose [mmol/L] × fasting insulin [mU/L]) × (mean OGTT glucose [mmol/L] × mean OGTT insulin [mU/L] [18,19].

The disposition index (DI) was calculated as the product of insulin secretion index and ISI: DI_30_ = ΔI30/ΔG30 × ISI [20] or DI = CIR × ISI [17], which indicates the insulin secretion function after the adjustment for insulin sensitivity.

### Statistical analysis

All statistical analyses were performed using the SUDAAN (version 10; Research Triangle Institute, NC, USA), as appropriate for the complex survey design. All calculations were weighted to represent the total Chinese adult population aged 20 years or older. Weights were calculated based on the data from the Chinese population of the year 2006 and the study sampling scheme, and took several features of the survey into account, including oversampling for female and urban residents, nonresponse, economic development, and other demographic or geographic differences between the sample and the total population [2]. The data are expressed as means with 95% confidence interval (95% CI) or median with the interquartile range. The difference between the group means and the between-group frequencies was tested using the PAIRWISE procedure in SUDAAN 10. The test for trend was performed with a polynomial contrast procedure. Variables that were not normally distributed were transformed logarithmically before the analysis. A multiple logistic regression analysis was used to examine the association between the family history of diabetes and the prevalence of diabetes, and the adjusted odd ratios were obtained. All *P* values were two-tailed, and a *P* value of < 0.05 was considered statistically significant.

## Results

### Subject disposition

The survey database collected information on a total of 46,239 subjects, including 18,419 men and 27,820 women (mean age 44.9 ± 13.7 years). Of these, 9958 subjects were excluded for incomplete or inaccurate report on the family history of diabetes. The present study focused on 36,281 subjects with a complete dataset including 14,263 men and 22,018 women. Out of the 36,281 subjects, 5443 subjects were classified into the risk category of FH1, accounting for a weighted percentage of 13.9% (95% CI: 13.2–14.5%); 643 subjects in the risk category of FH2, accounting for a weighted percentage of 1.6% (95% CI: 1.3–2.0%); and the remaining in the risk category of FH0. There were no significant differences in the mean age among the three family history risk categories of diabetes, i.e., 44.9 years for FH0 and FH1 and 45.1 years for FH2. The age of onset of diabetes was 1–2 years earlier in the groups with a positive family history of diabetes (FH1 and FH2) than in those of FH0.

### Family history risk categories of the subjects

With increasing family history risk category of diabetes, the levels of previous maximal weight, prevalence of central obesity, present BMI, systolic and diastolic blood pressure, fasting glucose and insulin, 2-h postloading glucose and insulin levels, total serum cholesterol levels, triglyceride levels, and low-density lipoprotein cholesterol levels all showed a significantly increasing trends (Table 1).

**Table 1:** General clinical characteristics of the Chinese population with positive or negative history of diabetes (adjusted for age and gender).

	FH0	FH1	FH2	*P*-value		
				FH1 *vs* FH0	FH2 *vs* FH0	FH1 *vs* FH2
Number	30,195	5443	643			
Gender (M/F)	11,951/18,244	2072/3371	240/403			
Prevalence of DM (%)	8.4	20.1	32.7	<0.0001	<0.0001	0.0003
	7.9, 8.9	18.2, 22.1	26.4, 39.7			
Age (years)	44.9	44.9	45.1	0.2834	0.1876	0.3892
	44.8, 44.9	44.8, 45.1	44.7, 45.5			
Age of diagnosis of DM (years)	45.3	43.9	44.6	0.0002	0.0409	0.2035
	45.1, 45.4	43.2, 44.6	43.9, 45.2			
Maximal weight (kg)	65.3	69.2	71.0	<0.0001	<0.0001	0.0075
	65.1, 65.6	68.6, 69.8	69.9, 72.2			
Smoking (%)	24.8	25.6	29.1	0.4312	0.1609	0.2841
	23.9, 25.6	23.7, 27.7	23.5, 35.5			
Drinking (%)	21.3	26.2	28.6	<0.0001	0.0171	0.4480
	20.5, 22.1	24.3, 28.1	23.0, 34.9			
Central obesity (%)	27.40	34.00	41.7000	<0.0001	<0.0001	0.0148
	26.6, 28.3	31.8, 36.4	36.1, 47.5			
Body mass index (kg/m^2^)	23.85	24.54	25.11	<0.0001	<0.0001	0.0204
	23.78, 23.92	24.34, 24.74	24.67, 25.56			
Systolic blood pressure (mmHg)	122.7	123.9	123.8	0.0158	0.2598	0.9289
	122.4, 123.1	123.0, 124.7	122.0, 125.6			
Diastolic blood pressure (mmHg)	77.8	78.6	80.6	0.0100	<0.0001	0.0039
	77.6, 78.1	78.1, 79.2	79.4, 81.8			
FPG (mmol/L)	5.2	5.8	6.5	<0.0001	<0.0001	0.0005
	5.2, 5.2	5.6, 6.0	6.2, 6.8			
PG2h (mmol/L)	6.8	8.1	9.6	<0.0001	<0.0001	0.0001
	6.8, 6.9	7.8, 8.4	8.9, 10.2			
FINS (mU/L)*	6.9	7.7	7.3	0.0006	0.0238	0.6814
	5.0, 9.6	5.5, 10.7	5.1, 10.7			
INS2h (mU/L)*	25.4	31.2	34.6	<0.0001	0.0006	0.8351
	15.2, 43.3	18.6, 54.9	19.0, 56.9			
TCHO (mmol/L)*	4.66	4.74	5.00	0.0024	<0.0001	0.0015
	4.03, 5.34	4.18, 5.37	4.42, 5.52			
TG (mmol/L)*	1.21	1.39	1.64	<0.0001	0.0004	0.2556
	0.85, 1.82	0.94, 2.07	0.98, 2.24			
LDLC (mmol/L)*	2.54	2.75	3.11	<0.0001	<0.0001	0.1305
	2.04, 3.09	2.28, 3.29	2.52, 3.37			
HDLC (mmol/L)*	1.25	1.25	1.25	0.4728	0.9045	0.8669
	1.07, 1.48	1.06, 1.48	1.05, 1.53			
ΔI30/ΔG30^a,^*	8.28	7.75	5.97	0.0132	0.8311	0.2763
	4.39, 15.50	3.91, 14.92	4.03, 12.53			
CIR*	750.8	688.2	461.9	0.0001	<0.0001	0.0039
	427.3, 1324.3	375.3, 1261.9	266.1, 954.5			
ISI*	136.3	116.9	115.5	<0.0001	<0.0001	0.0107
	92.7, 193.9	76.8, 165.6	73.2, 153.2			
HOMA-IR*	1.53	1.77	1.86	<0.0001	<0.0001	0.0086
	1.08, 2.25	1.22, 2.70	1.40, 2.98			
DI_30_^b,^*	1107.6	926.9	580.8	<0.0001	<0.0001	0.4604
	606.6,1888.3	450.9, 1654.6	397.4, 1296.9			
DI^c^ ^,^* (×10^3^)	104.1	84.1	48.5	<0.0001	<0.0001	0.0001
	60.7, 167.8	44.4, 142.9	24.2, 94.4			

Note: Variables with asterisk (*) are presented as the range of median to quartile, and other variables are presented as the mean with 95% CI.

^a^ ΔI30/ΔG30 = (INS30 - FINS)/(PG30 - FPG).

^b^ DI_30_ = ΔI30/ΔG30 × ISI.

^c^ DI = CIR × ISI.

ISI = 10,000/√(fasting plasma glucose [mmol/L] × fasting insulin [mU/L]) × (mean OGTT glucose [mmol/L] × mean OGTT insulin [mU/L]).

CI: confidence interval; CIR: corrected insulin response; DI: disposition index; DM: diabetes mellitus; FH0: negative family history of diabetes; FH1: moderate family history risk category of diabetes; FH2: high family history risk category of diabetes. FINS: fasting plasma insulin; FPG: fasting plasma glucose; HDLC: high-density lipoprotein cholesterol; HOMA-IR: homeostatic model assessment-estimated insulin resistance; INS2h: 2-h postload plasma insulin; INS30: serum insulin level at 30 min after OGTT; ISI: insulin sensitivity index; LDLC: low-density lipoprotein cholesterol; OGTT: oral glucose tolerance test; PG2h: 2-h postload plasma glucose; PG30: plasma glucose level at 30 min after OGTT; TCHO: total serum cholesterol; TG: serum triglyceride.

### Association between family history risk categories and prevalence of diabetes

The age- and gender-adjusted prevalence rates of diabetes were 8.4% (95% CI: 7.9–8.9%) in the risk category of FH0, 20.1% (95% CI: 18.2–22.1%) in the risk category of FH1, and 32.7% (95% CI: 26.4–39.7%) in the risk category of FH2, showing a statistically significant increasing trend (*P* < 0.0001) (Table 1). Further, the age and BMI were stratified to explore the association between the different family history risk categories and the prevalence of diabetes (Table 2). Results showed that in each level of age or BMI, the prevalence of diabetes significantly increased with the family history risk category (all *P* < 0.0001).

**Table 2:** Prevalence of diabetes in different family history risk categories in the Chinese population (%, means with 95%CI).

		FH0	FH1	FH2	*P* for trend
	20≤40	2.8	7.3	28.6	0.0001
		2.3, 3.3	5.7, 9.2	17.8, 42.5	
Age*	40≤60	10.1	21.4	30.4	<0.0001
(years)		9.3, 10.9	19.0, 24.1	24.4, 37.2	
	≥60	17.5	46.7	52.4	<0.0001
		15.5, 19.6	37.3, 56.4	36.2, 68.1	
	<25	6.1	16.9	33.0	<0.0001
		5.6, 6.8	14.6, 19.5	26.5, 40.3	
Body mass index**	25≤30	11.2	23.6	37.4	<0.0001
(kg/m^2^)		10.2, 12.3	20.5, 26.9	31.8, 43.4	
	≥30	17.4	26.7	41.0	0.0003
		14.5, 20.8	21.6, 32.5	29.5, 53.7	

* Gender-adjusted prevalence of diabetes.

** Age- and gender-adjusted prevalence of diabetes.

CI: confidence interval; FH0: negative family history of diabetes; FH1: moderate family history risk category of diabetes; FH2: high family history risk category of diabetes.

### Association between family history risk categories and insulin secretion and resistance

In comparison to those in the risk category of FH0, both ΔI30/ΔG30 and CIR continuously decreased in the risk categories of FH1 and FH2. The indicator for insulin sensitivity, ISI, also successively decreased in FH1 and FH2, while HOMA-IR, indicative of insulin resistance, showed an increase. After the adjustment for insulin sensitivity, the declining tendency in the insulin secretory function (described by DI and DI_30_) became more significant with the increasing family history risk category of diabetes (Table 1). Even if the glucose tolerance status was further divided into three levels (normal glucose tolerance, NGT; prediabetes; and diabetes), there remained significant declining trends in DI and DI_30_ of different glucose tolerance statuses (Table 3).

**Table 3:** Association between family history risk categories and insulin secretion and resistance.

	NGT				Prediabetes				DM			
	FH0	FH1	FH2	P for trend	FH0	FH1	FH2	P for trend	FH0	FH1	FH2	P for trend
ΔI30/ΔG30	9.29	9.57	9.60	0.0001	6.31	6.25	4.26	0.1737	3.15	3.35	3.22	0.2013
	5.04, 16.97	5.03, 17.27	5.37, 21.63		3.42, 11.51	3.54, 12.11	4.19, 6.29		1.64,7. 21	1.69, 5.64	1.51, 7.50	
CIR	860.8	890.0	917.8	0.2336	554.7	603.9	406.8	0.2741	258.7	252.5	195.5	0.0091
	521.8, 1487.39	517.4, 1499.4	504.8, 1515.6		326.6, 958.2	373.5, 1025.9	396.7, 558.4		142.0, 505.0	133.0, 507.7	88.6, 462.6	
HOMA-IR	1.42	1.55	1.70	<0.0001	1.77	2.01	1.40	0.1251	2.79	3.55	3.29	0.1860
	1.03,2.01	1.13, 2.16	0.94, 2.37		1.19, 2.64	1.32, 3.02	1.39, 2.25		1.67, 4.42	2.23, 5.91	2.00, 5.81	
ISI	148.9	134.7	122.9	<0.0001	107.8	89.8	117.4	0.0844	80.5	71.9	73.5	0.7144
	105.7, 208.2	97.2, 181.1	84.0, 193.3		72.2, 157.1	60.9, 138.2	92.7, 117.9		52.9, 122.1	44.1, 101.9	43.4, 132.1	
DI_30_	1320.4	1186.9	1256.2	0.4225	663.4	588.4	501.1	<0.0001	270.3	221.8	199.9	0.0010
	812.2, 2138.8	722.1, 1981.5	754.6, 2063.5		407.2, 994.9	361.9, 902.3	435.3, 555.8		144.0, 513.0	124.7, 395.4	125.9, 460.0	
DI (× 10^3^)	125.3	113.2	101.4	0.0001	59.4	52.2	48.2	0.0004	22.2	18.5	16.5	0.0001
	83.5, 129.6	74.9, 172.9	71.9, 177.5		40.9, 85.3	40.5, 78.2	40.9, 50.3		12.6, 37.9	9.3, 29.5	8.4, 25.1	

Note: Variables are presented as the range of median to quartile.

ΔI30/ΔG30 = (INS30 - FINS)/(PG30 - FPG); ISI = 10,000/√(fasting plasma glucose [mmol/L] × fasting insulin [mU/L]) × (mean OGTT glucose [mmol/L] × mean OGTT insulin [mU/L]); DI = CIR × ISI; DI_30_ = ΔI30/ΔG30 × ISI

CIR: corrected insulin response; DI: disposition index; DM: diabetes mellitus; FH0: negative family history of diabetes; FH1: moderate family history risk category of diabetes; FH2: high family history risk category of diabetes; FINS: fasting plasma insulin; FPG: fasting plasma glucose; HOMA-IR: homeostatic model assessment-estimated insulin resistance; INS30: serum insulin level at 30 min after OGTT; ISI: insulin sensitivity index; NGT: normal glucose tolerance; OGTT: oral glucose tolerance test; PG30: plasma glucose level at 30 min after OGTT.

**Figure 1:**
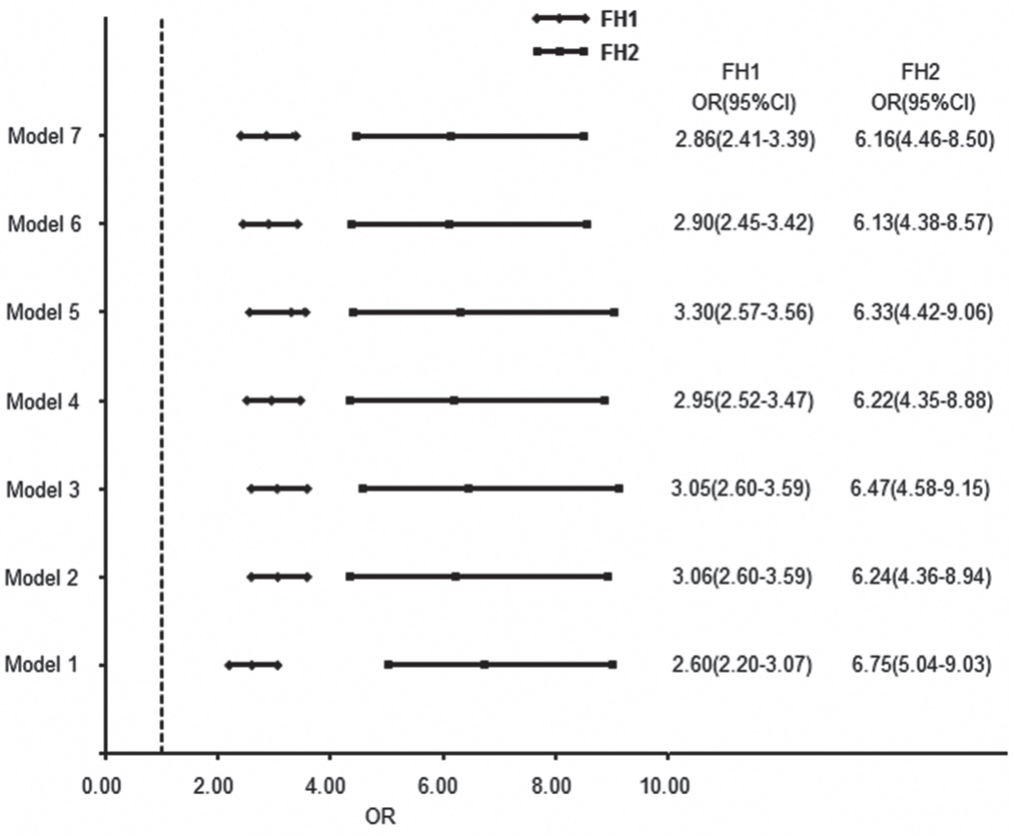
The association between family history risk category and prevalence of diabetes (OR with 95% CI). The ORs were calculated using logistic regression analysis with FH0 as the reference. Model 1: no adjustment for any variables; Model 2: adjusted by age and gender; Model 3: adjusted by age, gender, and systolic blood pressure; Model 4: adjusted by age, gender, systolic blood pressure, and geographic region (rural or urban); Model 5: adjusted by age, gender, systolic blood pressure, geographic region, and education level; Model 6: adjusted by age, gender, systolic blood pressure, geographic region, education level, and BMI; and Model 7: adjusted by age, gender, systolic blood pressure, geographic region, education level, BMI, and waist circumference. BMI: body mass index; CI: confidence interval; FH0: negative family history of diabetes; OR: odds ratio.

A logistic regression analysis was performed with or without adjustment for recognized risk factors for onset of diabetes (including age, gender, blood pressure, BMI, waist circumference, economic condition, and education level). Different models were used to calculate the odds ratios (ORs) of having diabetes in different family history risk categories (Fig. 1). Compared with those in the low risk category of FH0, ORs relatively increased to 2.60–3.30 in the moderate risk category of FH1 and 6.13–6.75 in the high risk category of FH2 (Fig. 1).

## Discussion

The present study involved a retrospective analysis of data from the 2007–2008 China National Diabetes and Metabolism Disorders Study involving 46,239 subjects. Out of these subjects, 36,281 subjects (14,263 men and 22,018 women) with a complete dataset were focused in this study. The results indicated that family history risk categories studied were found to have a significant association with prevalence of diabetes in the country. Hence, the results carry significant relevance in understanding the etiology and prevalence of diabetes among the Chinese population. The etiology of diabetes involves genetic and environmental factors as well as their complex interactions[4,5]. Substantial epidemiological research has confirmed that family history of diabetes, advanced age, obesity (especially abdominal obesity), physical inactivity, and abnormal lipid metabolism are independent risk factors for diabetes[2,3,6,7]. Individuals with a positive family history of diabetes experience 3- to 4-fold higher risks for this disease than those with a negative family history of diabetes [2,3,6–8], and the risk of developing diabetes is related to the type [8] and number of affected relatives [6–9].

In the nationwide cross-sectional study, results showed that the proportion of first-degree relatives with a positive family history of diabetes was up to 15.5% in the Chinese population. Of these, the proportion of at least two generations of the first-degree relatives with a positive family history of diabetes was 1.6%. The age of diagnosis of diabetes in those with a positive family history of diabetes was earlier than those with a negative family history of this disease. This study took the lead to classify the family history of diabetes into different risk categories in the Chinese population according to the generation number of the affected first-degree relatives. Additionally, this study demonstrated that with the increasing family history risk category of diabetes, a gradual increase was observed in the levels of diabetes-associated metabolic abnormalities including central obesity, BMI, blood pressure, and lipid levels. Moreover, the group with a positive family history of diabetes had higher ratios of smoking and drinking, to some extent reflecting a more negative lifestyle. Thus, it is not surprising that the prevalence of diabetes increased with the family history risk category of this disease.

As the age and BMI are strong risk factors for diabetes, this study also referred to the National Health and Nutrition Examination Survey of the United States (NHANES) [9] and analyzed the association between family history risk category and prevalence of diabetes after stratification of age and BMI into multiple levels. At each level of the age or BMI, the prevalence rate of diabetes continued to increase significantly with the family history risk category of this disease. The results of a further multivariate analysis showed that the hierarchical association between the family history risk category and the prevalence rate of diabetes was independent of some major risk factors for diabetes such as age, gender, BMI, and economic development. This conclusion is consistent with that of NHANES, despite certain difference between the definitions of family history risk category in both the studies.

Insulin resistance and abnormal insulin secretion are the most important prerequisites for the occurrence of type 2 diabetes. Some studies show that the first-degree relatives of type 2 diabetes have experienced insulin resistance even in the normal status of glucose tolerance [21–23], whereas other studies have presented different results and proposed that the first-degree relatives of diabetes mainly had abnormal insulin secretion [17,24,25]. The main reasons for the above contradiction are as follows: (1) The interrelation between insulin secretion and insulin sensitivity is not considered. The maintenance of glucose homeostasis depends on the balance of insulin secretion and insulin sensitivity. There is a hyperbolic equation between both the above-mentioned parameters, i.e., the product of insulin sensitivity and acute insulin response is a constant, commonly known as the glucose DI [26]. The DI is equivalent to the β-cell secretion function after the adjustment for insulin sensitivity. (2) Insulin secretion and insulin sensitivity are not estimated after the stratification of different glucose tolerance statuses because the levels of impaired insulin secretion and insulin resistance vary with different glucose tolerance types [27–29]. (3) The accuracy of estimating insulin secretion and insulin sensitivity using simple indices based on fasting and postloading blood glucose and insulin levels may vary in different glucose tolerance statuses [30], as confirmed by the results of this study.

In this study, through the overall analysis on the Chinese population, it was found that with the increasing family history risk category of diabetes, the insulin secretion indices both ΔI30/ΔG30 and CIR exhibited significant decreasing tendencies, while HOMA-IR showed progressive increases and ISI showed gradual declines. After the adjustment for insulin resistance, the insulin secretion indices DI_30_ and DI exhibited even more significant declining tendencies. Further, the population was divided into three groups according to the glucose tolerance status, i.e., NGT, prediabetes, and diabetes. Results showed that ΔI30/ΔG30 had a compensatory increase with the family history risk category in the NGT group, with no significant changes in the other two groups (prediabetes and diabetes). In contrast, CIR significantly declined with increasing family history risk category in the diabetes group. Both DI_30_ and DI had linear decreases with increasing family history risk category in all the three groups (NGT, prediabetes, and diabetes). The above-mentioned results indicate that the first-degree relatives of diabetes mainly had β-cell dysfunction, and that the higher the family history risk category, the more severe the β-cell dysfunction.

The present study also has its own limitations. First, in the 2007–2008 DMS database, information regarding the prevalence of diabetes in the first-degree relatives is incomplete, unknown, or missing for one-fifth of the subjects. During the in-depth analysis, these subjects were excluded, which might raise questions regarding the representativeness of the study data for the overall situation of the Chinese population. This is despite the fact that the general information was compared between the groups included and excluded, and found no significant differences in the major parameters related to diabetes (age, gender, BMI, waist circumference, blood pressure, and education level). Second, the 2007–2008 DMS database provides information on the prevalence of diabetes in the first-degree relatives but not in the second-degree relatives. Thus, in this study, the definition of family history risk category of diabetes may be restrictive. Finally, the family history of diabetes was self-reported by the subjects through a questionnaire survey, perhaps with reporting bias. The information obtained was not verified later, and the accuracy of reports was not validated through random sampling. However, a unified training of inquiry methods and techniques was provided for physicians or nurses involved in collecting information on the family history of diabetes; and only the first-degree relatives (parents, siblings, and children) who are closely associated with the subjects in their daily life were surveyed. Thus, it is reasonable to believe that the accuracy of reports on disease history is relatively high.

In conclusion, this study represents a large-sample representative study of the Chinese population. Results show that family history risk category of the first-degree relatives of diabetes not only significantly relates to the level of β-cell defects but also has a significant and independent rank correlation with the prevalence of diabetes in individuals. Risk assessment, screening, and prevention of diabetes should therefore consider the positive or negative as well as the risk category of family history of this disease.

## Supporting Information

S1 Appendix Members of the China National Diabetes and Metabolic Disorders Study Group. (DOC)

## References

[1] International Diabetes Federation (2013) IDF Diabetes Atlas, 6th edn. Brussels, Belgium: International Diabetes Federation 10.3390/jpm3040288

[2] Yang W, Lu J, Weng J, Jia W, Ji L, et al (2010) Prevalence of diabetes among men and women in China. N Engl J Med 362: 1090–1101. 10.1056/NEJMoa0908292 20335585

[3] Xu Y, Wang L, He J, Bi Y, Li M, et al (2013) Prevalence and control of diabetes in Chinese adults. JAMA 310: 948–959. 10.1001/jama.2013.168118 24002281

[4] Moonesinghe R, Ioannidis JP, Flanders WD, Yang Q, Truman BI, et al (2012) Estimating the contribution of genetic variants to difference in incidence of disease between population groups. Eur J Hum Genet 20:831–836. 10.1038/ejhg.2012.15 22333905 PMC3400729

[5] Temelkova-Kurktschiev T, Stefanov T (2012) Lifestyle and genetics in obesity and type 2 diabetes. Exp Clin Endocrinol Diabetes 120:1–6. 10.1055/s-0031-1285832 21915815

[6] Grill V, Persson G, Carlsson S, Norman A, Alvarsson M, et al (1999) Family history of diabetes in middle-aged Swedish men is a gender unrelated factor which associates with insulinopenia in newly diagnosed diabetic subjects. Diabetologia 42: 15–23. 10027572 10.1007/s001250051106

[7] Hariri S, Yoon PW, Qureshi N, Valdez R, Scheuner MT, et al (2006) Family history of type 2 diabetes: a population-based screening tool for prevention? Genet Med 8: 102–108. 16481893 10.1097/01.gim.0000200949.52795.df

[8] Meigs JB, Cupples LA, Wilson PW (2000) Parental transmission of type 2 diabetes: the Framingham Offspring Study. Diabetes 49: 2201–2207. 11118026 10.2337/diabetes.49.12.2201

[9] Valdez R, Yoon PW, Liu T, Khoury MJ (2007) Family history and prevalence of diabetes in the U.S. population: the 6-year results from the National Health and Nutrition Examination Survey (1999–2004). Diabetes Care 30: 2517–2522. 17634276 10.2337/dc07-0720

[10] Kim DJ, Cho NH, Noh JH, Lee MS, Lee MK, et al (2004) Lack of excess maternal transmission of type 2 diabetes in a Korean population. Diabetes Res Clin Pract 65: 117–124. 15223223 10.1016/j.diabres.2003.11.020

[11] Franks PW (2010) Diabetes family history: a metabolic storm you should not sit out. Diabetes 59: 2732–2734. 10.2337/db10-0768 20980473 PMC2963529

[12] Pan XR, Yang WY, Li GW, Liu J (1997) Prevalence of diabetes and its risk factors in China, 1994 National Diabetes Prevention and Control Cooperative Group. Diabetes Care 20: 1664–1669. 9353605 10.2337/diacare.20.11.1664

[13] Joint Committee for Developing Chinese guidelines on Prevention and Treatment of Dyslipidemia in Adults (2007) Chinese guidelines on prevention and treatment of dyslipidemia in adults. Zhonghua Xin Xue Guan Bing Za Zhi 35: 390–419.(in Chinese) 17711682

[14] Bao Y, Lu J, Wang C, Yang M, Li H, et al (2008) Optimal waist circumference cutoffs for abdominal obesity in Chinese. Atherosclerosis 201: 378–384. 10.1016/j.atherosclerosis.2008.03.001 18417137

[15] Department of Noncommunicable Disease Surveillance (1999) Definition, diagnosis and classification of diabetes mellitus and its complications: report of a WHO consultation. Part 1. Diagnosis and classification of diabetes mellitus. Geneva: World Health Organization

[16] Haffner SM, Miettinen H, Gaskill SP, Stern MP (1996) Decreased insulin action and insulin secretion predict the development of impaired glucose tolerance. Diabetologia 39: 1201–1207. 8897008 10.1007/BF02658507

[17] Isomaa B, Forsen B, Lahti K, Holmstrom N, Waden J, et al (2010) A family history of diabetes is associated with reduced physical fitness in the Prevalence, Prediction and Prevention of Diabetes (PPP)-Botnia study. Diabetologia 53: 1709–1713. 10.1007/s00125-010-1776-y 20454776

[18] Matthews DR, Hosker JP, Rudenski AS, Naylor BA, Treacher DF, et al (1985) Homeostasis model assessment: insulin resistance and beta-cell function from fasting plasma glucose and insulin concentrations in man. Diabetologia 28: 412–419. 3899825 10.1007/BF00280883

[19] Matsuda M, DeFronzo RA (1999) Insulin sensitivity indices obtained from oral glucose tolerance testing: comparison with the euglycemic insulin clamp. Diabetes Care 22: 1462–1470. 10480510 10.2337/diacare.22.9.1462

[20] Muniyappa R, Lee S, Chen H, Quon MJ (2008) Current approaches for assessing insulin sensitivity and resistance in vivo: advantages, limitations, and appropriate usage. Am J Physiol Endocrinol Metab 294: E15–26. 17957034 10.1152/ajpendo.00645.2007

[21] Ishikawa M, Pruneda ML, Adams-Huet B, Raskin P (1998) Obesity-independent hyperinsulinemia in nondiabetic first-degree relatives of individuals with type 2 diabetes. Diabetes 47: 788–792. 9588451 10.2337/diabetes.47.5.788

[22] Warram JH, Martin BC, Krolewski AS, Soeldner JS, Kahn CR (1990) Slow glucose removal rate and hyperinsulinemia precede the development of type II diabetes in the offspring of diabetic parents. Ann Intern Med 113: 909–915. 2240915 10.7326/0003-4819-113-12-909

[23] Gulli G, Ferrannini E, Stern M, Haffner S, DeFronzo RA (1992) The metabolic profile of NIDDM is fully established in glucose-tolerant offspring of two Mexican-American NIDDM parents. Diabetes 41: 1575–1586. 1446799 10.2337/diab.41.12.1575

[24] Goran MI, Coronges K, Bergman RN, Cruz ML, Gower BA (2003) Influence of family history of type 2 diabetes on insulin sensitivity in prepubertal children. J Clin Endocrinol Metab 88: 192–195. 12519851 10.1210/jc.2002-020917

[25] van Haeften TW, Dubbeldam S, Zonderland ML, Erkelens DW (1998) Insulin secretion in normal glucose-tolerant relatives of type 2 diabetic subjects. Assessments using hyperglycemic glucose clamps and oral glucose tolerance tests. Diabetes Care 21: 278–282. 9539996 10.2337/diacare.21.2.278

[26] Utzschneider KM, Prigeon RL, Carr DB, Hull RL, Tong J, et al (2006) Impact of differences in fasting glucose and glucose tolerance on the hyperbolic relationship between insulin sensitivity and insulin responses. Diabetes Care 29:356–362. 16443887 10.2337/diacare.29.02.06.dc05-1963

[27] Hanefeld M, Koehler C, Fuecker K, Henkel E, Schaper F, et al (2003) Insulin secretion and insulin sensitivity pattern is different in isolated impaired glucose tolerance and impaired fasting glucose: the risk factor in Impaired Glucose Tolerance for Atherosclerosis and Diabetes study. Diabetes Care 26: 868–874. 12610051 10.2337/diacare.26.3.868

[28] Weyer C, Bogardus C, Pratley RE (1999) Metabolic characteristics of individuals with impaired fasting glucose and/or impaired glucose tolerance. Diabetes 48: 2197–2203. 10535454 10.2337/diabetes.48.11.2197

[29] Davies MJ, Raymond NT, Day JL, Hales CN, Burden AC (2000) Impaired glucose tolerance and fasting hyperglycaemia have different characteristics. Diabet Med 17: 433–440. 10975211 10.1046/j.1464-5491.2000.00246.x

[30] Hanson RL, Pratley RE, Bogardus C, Narayan KM, Roumain JM, et al (2000) Evaluation of simple indices of insulin sensitivity and insulin secretion for use in epidemiologic studies. Am J Epidemiol 151: 190–198. 10645822 10.1093/oxfordjournals.aje.a010187

