# Supplementary material for: Association between Family History Risk Categories and Prevalence of Diabetes in Chinese Population
Source: PLoS One. 2015 Feb 9;10(2):e0117044. doi: 10.1371/journal.pone.0117044 (PMC4321835; doi:10.1371/journal.pone.0117044)
Supplement: S1 Appendix — (DOC) [file pone.0117044.s001.doc]

Appendix S1. China National Diabetes and Metabolic Disorders Study (DMS) Group.
Consulting Members: 
Kunsan Xiang, Jialun Chen, Changyu Pan, Zuzhi Fu
DMS Field Centers (all field centers contributed to this work equally)  
1. China-Japan Friendship Hospital, Beijing, China: Wenying Yang (principal investigator), Jianzhong Xiao, Zhaojun Yang, Danjie Ruan (Huairou 1st Hospital),Yufeng Li (Pinggu Hospital), Shi Bu, Hongliang Li, Peng Wang, Xueli Liu, and Zhiyun Xiao. 
2. Chinese PLA General Hospital, Beijing, China: Juming Lu (principal investigator), Jingtao Dou, Nan Jin, Jing Li, Shuyu Wang, Baojing Zhang, and Liguang Dong.
3. Third Hospital, Sun Yat-sen University, Guangzhou, China: Jianping Weng (principal investigator), Longyi Zeng, Panwei Mu, Yanhua Zhang, Qiuqiong Yu, Keyi Lin, Yan Sun, and Xiaodong Xu. 
4. Shanghai Jiaotong University Affiliated Sixth People's Hospital, Shanghai, China: Weiping Jia (principal investigator), Xuhong Hou, Xiaojing Ma, Huijuan Lu, Dajing Zou (Changhai Hospital), Renming Hu (Huashan Hospital), Huilin Gu (Huayang Communities), Youhua Yao (Linfen Communities), Weizhen Shen (Pengpu Communities), Junbin Huang (Tianmu Communities), and Zhijian Pan (Anting Communities) 
5. Peking University Peoples' Hospital, Beijing, China: Linong Ji (principal investigator), Xianghai Zhou, Xueyao Han, Xiuying Zhang, Yingying Luo, Xiuqing Sun, Dong Zhao (Luhe Hospital), Huifang Xing (Mentougou Hospital), Lei Liu (Haidianqu), and Xinquan Zhang (Jingyuan Hospital) 
6. The First Affiliated Hospital, Chinese Medical University, Liaoling, China: Zhongyan Shan (principal investigator), Yaxin Lai, Xiaochun Teng, Ling Shan, Jiani Wang, Liangfeng Shi, Sen Wang, Li Lu, Fengwei Jiang, and Beibei Wang. 
7. Shanxi Province People's Hospital, Shanxi, China: Jie Liu (principal investigator), Ling Hu, and Yuying Hou (Shan Xi Medical University). 
8. West China Hospital, Sichuan University, Sichuan, China: Haoming Tian (principal investigator), Xingwu Ran, Yan Ren, and Hongling Yu; the First People's Hospital of Liangshan Yi Nationality Autonomy District, Xichan City (Lisheng Cao, Hualin Lu, Xiaohua Xie); Yulin Community Health Center of Wuhou District, Chengdu (Xilian Gao); and the First Hospital of Longquyi District, Chengdu (Zhong Li). 
9. Xijing Hospital, Fourth Military Medical University, Shaanxi, China: Qiuhe Ji (principal investigator), Fei Chen, and Yaping Zhang. 
10. The Affiliated Drum Tower Hospital of Nanjing University Medical School, Jiangsu, China: Dalong Zhu (principal investigator), Yun Hu, Guoyu Tong, Ning Xu, Jinluo Cheng, Junjian Chen, Fei Wang, and Jiong Pei. 
11. Xinjiang Uygur Autonomous Region's Hospital, Xingjiang, China: Jiapu Ge (principal investigator), Wan Yi, Rong Wang (Bayi Steel's Hospital), Gang Han, Huijie Mu (Habahe County Hospital), and Maimaiti Aireti ( Moyu County Hospital). 
12. Fujian Provincial Hospital, Fujian, China: Lixiang Lin (principal investigator), Gang Chen, Jingxin Zhao, Shuyu Yang, and Mingzhu Lin (Xiamen 1st Hospital), Yadong Zhang, Fengyuan Zhu (Sanming 1st Hosptial), Mei Tu (Longyan 1st Hospital), Shanghua Xu (Nanping 1st Hospital), and Weihong Lin (Jiangle County Hospital). 
13. Qilu Hospital of Shandong University, Shandong, China: Li Chen (principal investigator), Yu Sun, Xiaolin Dong (Jinan Central Hospital), Yiling Fu, Kehua Zhou, Jinbo Liu, Zhenzuo Li, Yuxin Xu, Peng Lin, and Wenjuan Li. 
14. Peking University First Hospital, Beijing, China: Xiaohui Guo (principal investigator), Junqing Zhang, Aimei Dong, Dongming Huang and Changchun Xue (Daxinqu Hospital) and Junqing Liu (Shichahai Community). 
15. Henan Province People's Hospital, Henan, China: Zhigang Zhao (principal investigator), Guijun Qin, Yong Yan, Peiyu Yao, and Qinchu Li. 
16. Haerbin Medical University Second Hospital, Heilongjiang, China: Qiang Li (principal investigator), Kaiting Chen, Nannan Wu, Yan Feng, Xiaoying Liu, and Guozhong Li. 
17. Xiangya Second Hospital, Hunan, China: Zhiguang Zhou (principal investigator), Weili Tang, Qiong Feng, Yuju Qin, Xinwen Qiu, Diaoxiang Xiao, Aiping Qin, Bin Liao, Zifang Gao, and Liang Xiang.
Statistics Analysis: 
Guangliang Shan, Peking Union Medical College, Beijing, China 
Wenying Yang, Jianzhong Xiao and Zhaojun Yang, China-Japan Friendship Hospital, Beijing, China 
Jiang He, Chung-Shiuan Chen, Tulane University School of Public Health and Tropical Medicine, New Orleans, LA, USA 
